# Co-occurrence of dual carbapenemases KPC-2 and OXA-48 with the mobile colistin resistance gene *mcr-9.1* in *Enterobacter xiangfangensis*

**DOI:** 10.3389/fcimb.2022.960892

**Published:** 2022-08-17

**Authors:** Yancheng Yao, Swapnil Doijad, Jane Falgenhauer, Judith Schmiedel, Can Imirzalioglu, Trinad Chakraborty

**Affiliations:** ^1^ Institute of Medical Microbiology, Justus Liebig University Giessen, Giessen, Germany; ^2^ German Center for Infection Research (DZIF), Partner Site Giessen-Marburg-Langen, Justus-Liebig University Giessen, Giessen, Germany; ^3^ Institute of Medical Microbiology, University Hospital Giessen, Giessen, Germany

**Keywords:** *Enterobacter*, *xangfangensis*, extreme-drug resistance, mobile colistin resistance, MCR-9.1, dual carbapenemases KPC-2 and OXA-48, plasmid

## Abstract

Bacterial infections with the genus *Enterobacter* are notoriously difficult to treat and often associated with resistance to penicillin, aminoglycosides, fluoroquinolones, and third-generation cephalosporins. Also, *Enterobacter* species have emerged as the third most common hosts for carbapenemases worldwide, forcing the use of colistin as a “last-resort” antibiotic for the treatment. Studies on the population structure of the genus *Enterobacter* repeatedly detect *E. xiangfangensis* as a common clinical species present worldwide. Here, we report on the characteristics of an extreme drug-resistant *E. xiangfangensis* isolate va18651 (ST88), obtained from a cervical swab of an expectant mother. The isolate was resistant to almost all the classes of antibiotics tested, including β-lactams (viz., penicillins, carbapenems, cephalosporin, monobactams, and their combinations), quinolone, aminoglycosides, and sulfonamide/dihydrofolate reductase inhibitor, and exhibited heteroresistance towards colistin. Analysis of its complete genome sequence revealed 37 antibiotic resistance genes (ARGs), including *mcr-9.1*, *bla_KPC-2_*, and *bla_OXA-48_*, encoded on three of the four different plasmids (cumulative plasmidome size 604,632 bp). An unusually high number of plasmid-based heavy metal resistance gene (HRG) clusters towards silver, arsenate, cadmium, copper, mercury, and tellurite were also detected. Virulence genes (VGs) for the lipopolysaccharide and capsular polysaccharide structures, iron acquisition (*iroBCDEN*, *ent/fep/fes*, *sitABCD*, *iut*, and *fur*), and a type VI secretion system, together with motility genes and Type IV pili, were encoded chromosomally. Thus, a unique combination of chromosomally encoded VGs, together with plasmid-encoded ARGs and HRGs, converged to result in an extreme drug-resistant, pathogenic isolate with survival potential in environmental settings. The use of a disinfectant, octenidine, led to its eradication; however, the existence of a highly antibiotic-resistant isolate with significant virulence potential is a matter of concern in public health settings and warrants further surveillance for extreme drug-resistant *Enterobacter* isolates.

## Introduction

The genus *Enterobacter* of the bacterial order *Enterobacterales* comprises environmental and clinical species (Davin-Regli et al., 2019). Clinical isolates of *Enterobacter* spp. are primarily opportunistic pathogens (Sanders and Sanders, 1997), involved mainly in hospital-associated infections of the urinary and respiratory tract as well as bloodstream infections (Kang et al., 2004; Ramirez and Giron, 2021). Due to similarities in the taxonomically relevant characters, isolates of different *Enterobacter* species have been misidentified as “*E. cloacae*” or “*Enterobacter* species” or “*Enterobacter cloacae* complex (ECC)”, and remained unrecognized with respect to their true species nomenclature (De Florio et al., 2018; Wu et al., 2020; Godmer et al., 2021). With the introduction of the high-resolution tools based on whole-genome sequencing in bacterial taxonomy, the precise delineation of bacterial species became possible (Hugenholtz et al., 2021). The genomically revised taxonomic structure of *Enterobacter* revealed *Enterobacter xiangfangensis* (also referred to as *Enterobacter hormaechei* subspecies *xiangfangensis*) as a common pathogenic species worldwide (Chavda et al., 2016; Peirano et al., 2018; Sutton et al., 2018; Wu et al., 2020; Cho et al., 2021; Wu et al., 2021).

*Enterobacter* species are frequently resistant to first-line antibiotics such as third-generation cephalosporin, penicillin, aminoglycosides, and quinolones, and fourth-generation cephalosporin and carbapenems are currently the most attractive therapeutic options (Davin-Regli et al., 2019). However, for the last 15 years, carbapenem resistance has been increasingly reported from *Enterobacterales* (Potter et al., 2016), with isolates of *Enterobacter* ranked among the top three in this group (Cerqueira et al., 2017). The most predominant carbapenemase type found in *E. xiangfangensis* was NDM, followed by VIM, KPC, OXA-48, and IMP (Peirano et al., 2018), while GIM-1, GIM-2, and IMI-9 were observed only sporadically (Wendel and MacKenzie, 2015; Di Luca et al., 2016; Wendel et al., 2018). Mobile genetic elements such as plasmids, particularly insertion sequence (IS) elements, were observed as a major determinant for the spread of these carbapenemase genes (Chavda et al., 2016; Potter et al., 2016).

For the “last-resort antibiotic”, such as colistin, *Enterobacter* species have been considered to be susceptible (WHO, 2018; WHO, 2021). Nevertheless, certain phylogenomic groups of this species exhibit colistin heteroresistance often leading to treatment failures as such isolates may initially be classified as being susceptible (Guérin et al., 2016; Mushtaq et al., 2020). A recent study shows that PhoPQ-dependent regulation of the *arnBCADTEF* gene cassette for transfer of 4-amino-4-deoxy-l-arabinose (l-Ara4N) to lipid A underlies colistin heteroresistance and resistance (Kang et al., 2019).

A trend of cumulative mobile resistance genes has been noted among members of *Enterobacterales*, including isolates of *Enterobacter* (Wang et al., 2019; Yang et al., 2022). Isolates carrying multiple resistance genes against different classes of antibiotics are increasingly reported (Chavda et al., 2019; Li et al., 2019). These genes may be juxtaposed within a single cassette, in different combinations, or on different plasmids within a single isolate. Co-existence of multiple resistance genes engenders extreme drug resistance and treatment of these infections are protracted with few therapeutic options remaining. Surveillance of such extensive or extreme drug-resistant pathogens and respective resistance genes harboring genetic elements is important to understand the epidemiology of dissemination and develop control strategies.

The SurvCARE study monitors the incidence of carbapenem-resistant *Enterobacterales* (CRE) in patients admitted in hospitals across the state of Hessen in Germany. During the surveillance (period 2017–2019), we noticed an overall increase in the number of the two-carbapenemase-carrying CRE isolates from 1.3% (1/79) in 2017, to 4.4% (5/113) in 2018, to 5.6% (9/162) in 2019, and detected an extreme drug-resistant (XDR) *E. xiangfangensis* isolate va18651, which was subsequently found, following whole-genome sequence analysis, to carry two carbapenemases, i.e., KPC-2 and OXA-48, as well as the mobile colistin resistance *mcr-9* gene. Further analysis reveals that the extreme drug resistance capability is plasmid-based, attained through the acquisition of four different plasmids, three of which carried 37 different antibiotic resistance genes that included two different carbapenemase genes *bla*_KPC-2_ and *bla*_OXA-48_ as well as the mobile colistin resistance gene *mcr-9.1*.

## Materials and methods

### Isolate, identification, and antibiotic resistance testing

During the 3-year surveillance study on carbapenem resistance (SurvCARE Hesse), the isolate va18651 was obtained from a cervical swab of an expectant mother during a routine checkup in November 2018. As a routine process, the swab samples were streaked on the Columbia blood and MacConkey agar plates. Colonies grown after overnight incubation at 37°C were randomly selected and identified by matrix-assisted laser desorption/ionization mass spectrometry (MALDI-TOF MS) (Vitek MS, bioMérieux, Nürtingen, Germany). The antimicrobial susceptibility testing was performed by using commercial MICRONAUT MIC-Strip (MERLIN Diagnostika GmbH, Bornheim, Germany), as well as by using cation-adjusted Mueller-Hinton broth 2. Results were interpreted based on the criteria of the European Committee on Antimicrobial Susceptibility Testing (Version 12.0). *E. coli* DH10β that exhibits an MIC of 2 against colistin was used as control. Colistin heteroresistance assays were performed by population analysis profiling as described earlier (Guérin et al., 2016).

### Whole-genome sequencing and bioinformatics analysis

The genome sequence of va18651 was obtained using PacBio SMRT sequencing technology using PacBio RSII machine (Pacific Biosciences, Menlo Park, CA, USA). The reads generated were assembled using the SMRT-Link Microbial Assembler 10.1.0.

The whole-genome sequence-based identification was carried out by calculating the average nucleotide identity (ANI) by BLAST using the JSpecies v1.2.1 tool (Richter et al., 2009) and by *in silico* DNA–DNA hybridization using the genome-to-genome distance calculator (formula 2) (Meier-Kolthoff et al., 2013) with the type strains of 23 *Enterobacter* species known as of May 2022 (Cho et al., 2021).

The assembled genome was annotated by the bacteria-specific annotation pipeline Bakta (Schwengers et al., 2021) and refined manually by using well-annotated reference genomes. The Multi-Locus Sequence Types (MLST), plasmid incompatibility (Inc) groups, plasmid MLST (pMLST), and acquired antibiotic resistance genes were identified using the Center for Genomic Epidemiology platform (https://cge.cbs.dtu.dk/services/) and the PubMLST database (https://pubmlst.org; https://bigsdb.pasteur.fr/cgi-bin/bigda.pl?db.), as described previously (Yao et al., 2021). Phylogenetic comparative genomics was performed based on single-nucleotide polymorphism (SNP) using Harvest Suite (ParSNP) (Treangen et al., 2014). The virulence genes were predicted by BLASTN against the VFDB database (Chen et al., 2016). The BLAST Ring Image Generator (BRIG) was employed to perform multiple comparisons of complete plasmid sequences available at the National Center for Biotechnology Information (NCBI), and circular maps were generated (Alikhan et al., 2011). To annotate the genetic contexts surrounding *bla*_KPC-2_, *bla*_OXA-48_, and *mcr-9.1* and mobile elements, Galileo AMR of ARC Bio was used (Partridge and Tsafnat, 2018). Distribution of the virulence genes was depicted on the circular genome using Circos v0.69.

To compare with other ST88 isolates, whole-genome sequences of 3,246 non-repetitive isolates (<1,000 contigs and >3 Mb assembly size) listed under the genus *Enterobacter* were downloaded from NCBI using e-utilities. These isolates were selected after reconfirming them as *bona fide Enterobacter* species using the OGRI tool as mentioned for va18651, and isolates identified to be ST88 were further studied.

### Data availability

The complete genome sequences of the *E. xiangfangensis* va18651 is available in public genome sequence databases with the accession numbers CP097342 (Chromosome), CP097343 (plasmid p1-va18651), CP097344 (p2-va18651), CP097345 (p3-va18651), and CP097346 (p4-va18651) within the BioProject PRJNA837392.

## Results

### Identification of the isolate va18651

**Figure 1:**
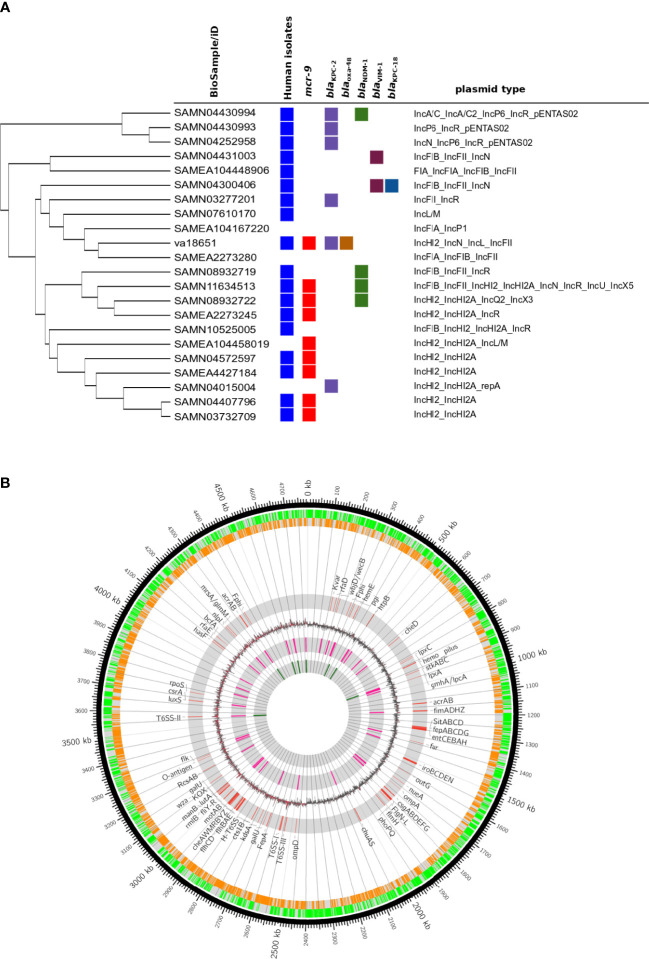
Genomic features of extensive drug-resistant *Enterobacter xiangfangensis* va18651. **(A)** Phylogenomic comparison of the va18651 to publicly available genomes of 21 ST88 isolates. The va18651 encoded mcr-9, blaKPC-2, and blaOXA-48 genes on three different plasmids with type IncHI2, IncN, and IncL, respectively. **(B)** Chromosomal features of *Enterobacter xiangfangensis* isolate va18651. The innermost to outermost (gray) circles indicate the respective locations of rRNA, tRNA, GC-skew, virulence genes, forward–reverse genes, and an ideogram indexing base pairs. The isolate va18651 carried all virulence genes on the chromosome while almost all antibiotic resistance genes were found on the plasmid.

Isolate va18651 was initially identified as a member of the “*Enterobacter* cloacae complex” using MALDI-TOF MS. Based on genome sequencing analysis, the average nucleotide identity (ANI) and *in silico* DNA–DNA hybridization scores (isDDH) were >95% (97.02%) and >70% (75.9%) as compared to type strain of *E. xiangfangensis* LMG 27195, respectively, confirming that va18651 is a member of the species *E. xiangfangensis*. The va18651 was sequence-typed to the clonal group ST88. Phylogenomic comparison to publicly available genomes revealed 21 ST88 isolates with comparable features for plasmid and antibiotic resistance genes (detailed below) (**Figure 1A**).

### The antimicrobial phenotype of the va18651

Broth microdilution methods indicated that the isolate va18651 was resistant to a large number of antibiotics (**Table 1**). The isolate showed the following MICs to the β-lactams tested: piperacillin (MIC > 32 µg/ml), piperacillin/tazobactam (MIC > 128/4 µg/ml), cefepime (MIC > 8 µg/ml), ceftazidime (MIC > 32 µg/ml), ceftazidime/avibactam (MIC ≤ 1/4 µg/ml), meropenem (MIC = 16 µg/ml), imipenem (MIC = 4 µg/ml), and aztreonam (MIC > 16 µg/ml). These results display that the isolate is resistant towards almost all β-lactams with the exception of ceftazidime-avibactam. The imipenem MIC leads to the breakpoint category susceptible under increased exposure according to EUCAST standards. However, as the MIC is at the upper limit of this category and the presence of carbapenemases was verified by WGS, this substance should not be used as a therapeutic agent for this isolate. This isolate was also phenotypically resistant to fluoroquinolone (ciprofloxacin), aminoglycosides (gentamicin and tobramycin), and sulfonamides (trimethomprim/sulfamethoxazole), but not resistant towards fosfomycin and colistin (**Table 1**). However, population analysis profiling for colistin showed a heteroresistance frequency (number of isolates grown on LB agar containing colistin compared to number of isolates grown on normal LB agar) of 1.3 ± 0.12% and 0.55 ± 0.04%, in the presence of 8 and 32 µg/ml of colistin, respectively. Given that the isolate may exhibit inherent low-level heteroresistance towards colistin, we repeated the broth-microdilution assay six times. In two of the assays, the isolate exhibited an MIC of 64 µg/ml toward colistin.

**Table 1:** Antimicrobial susceptibility of *Enterobacter xiangfangensis* va18651.

Antimicrobial	MIC (µg/ml)	Interpretation
**Aminoglycosides**		
Amikacin	8	S
Gentamicin	32	R
Tobramycin	16	R
**β-lactams**		
Piperacillin	>32	R
Piperacillin/Tazobactam	>128/4	R
Aztreonam	>16	R
Ceftazidime	>32	R
Cefepime	>8	R
Ceftazidime/Avibactam	≤1/4	S
Imipenem	4	I
Meropenem	16	R
**Fluoroquinolones**		
Ciprofloxacin	2	R
Levofloxacin	1	I
**Fosfomycin**		
Fosfomycin	≤16	S
**Sulfanilamide**		
Trimethomprim/sulfamethoxazole	>8/152	R
**Polymyxins**		
Colistin	0.25 64*	S R

In the case of colistin, skip-well phenomenon was noted, indicating heteroresistance capability (*). The antimicrobial susceptibility was performed with a broth microdilution assay and results were interpreted according to EUCAST criteria (version 12.0).

### Plasmidome and antimicrobial resistance determinants of the va18651

The general genomic features of va18651 are listed in **Table 2**. Based on long-read data, the genome of va18651 was assembled into five complete (i.e., circularized) contigs, including a 4,785,021-bp chromosome with a GC content of 55.5%, which encoded 4,433 predicted CDSs including a β-lactamase gene *bla*_ACT-7_ and 86 tRNAs, 25 rRNAs, and 83 ncRNAs (**Table 2**, **Figure 1B**) and four distinct plasmids that resulted in a plasmidome with a cumulative length of 604,632 bp (**Table 2**).

**Table 2:** Genomic features of *Enterobacter xiangfangensis* va18651.

Structure	Length(bp)	GC (%)	No. of CDS	Antimicrobial resistance genes	MLST/Inc type (pMLST)	Accession no.
Chromosome	4,785,021	55.51	4,433	*bla*_ACT-7,_ *phoPQ*-arnBCDATEF*	ST-88	CP097342
Plasmid						
p1-va18651	307,415	47.89	351	*mcr-9, bla*_SHV-12_ *, bla*_TEM-1B_ *, ere(A)*, *qacEΔ-1 (2x), catA2, tet(D), aadA2b, qnrA1, aac(6´)-Ib-cr, strA, strB, aac(6´)-Ib3, aac(6´)-IIc, sul1(3x), sul2, dfrA19*	IncHI2(pST1):: pKC-CAV1321	CP097343
p2-va18651	79,326	52.68	100	*bla*_KPC-2_ *, bla*_TEM-1B_ *,bla*_OXA-1_ *, aac(3)-IId*, *aac(6´)-Ib-cr, strA, strB, catB3, qacEΔ-1, ARR-3, mph(A), qnrB2, sul1 (2x), dfrA19*	IncN (pST15)	CP097344
p3-va18651	63,589	51.23	85	*bla*_OXA-48_	IncL	CP097345
p4-va18651	154,302	51.46	174	Not detected	IncFII(pECLA)	CP097346

A total of 36 (25 non-duplicated) genes were observed to be located on the plasmids. * The PhoPQ-dependent 4-amino-4-deoxy-l-arabinose addition to lipid A may result in the colistin heteroresistance in *Enterobacter cloacae* (Kang et al., 2019). The complete genome sequence revealed a chromosome and four plasmids.

**Figure 2:**
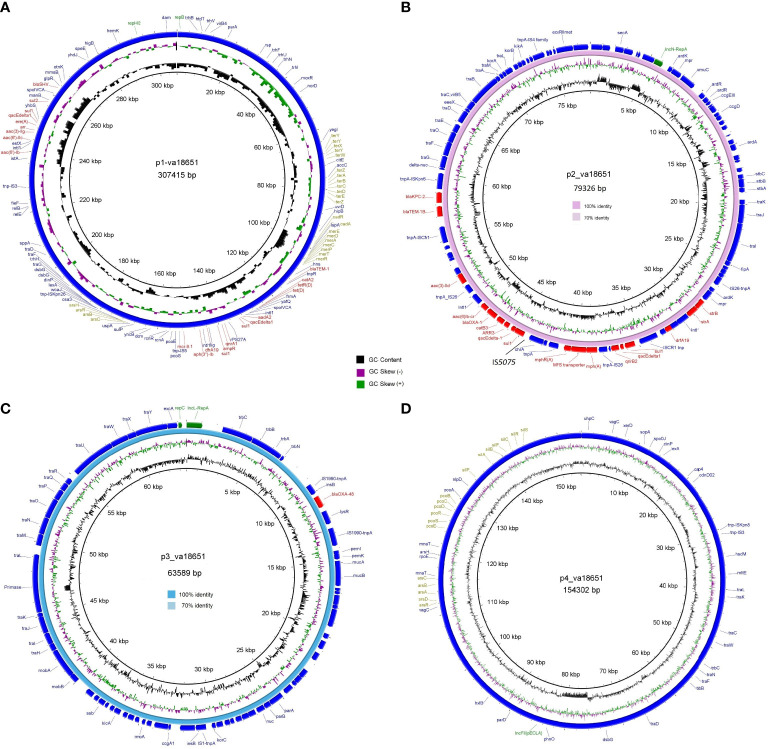
Circular genetic maps of the plasmids p1-va18651 **(A)**, p2-va18651 **(B)**, p3-va18651 **(C)**, and p4-va18651 **(D)** from the *E xiangfangensis* isolate va18651. Plasmid replicons, antimicrobial resistance, and heavy metal resistance are marked in green, red, and olive green, respectively. B depicts the BRIG comparison of p2-va18651 with pCP13069KPC2 (VKMZ0100118.1) and C displays the comparison of p3-va18651 with pOXA-48_1639 (LR025105).

The mobile colistin-resistance-gene *mcr-9.1*-containing plasmid, p1-va18651 was the largest plasmid carried by the *E. xiangfangensis* va18651 with 307,415 bp in length and a GC content of 47.9%. This mega-multi-replicon plasmid resulted from the fusion of an IncHI2(pST1) and the pKC-CAV1321 plasmids and encoded for a total of 351 predicted CDSs. The p1-va18651 also carried antibiotic resistance genes to β-lactams (*bla*_SHV-12_ and *bla*_TEM-1B_), aminoglycosides [*aadA2b, aac(6´)-Ib3, aac(6´)-IIc, strA*, and *strB*], fluoroquinolones [*qnrA1* and *aac(6´)-Ib-cr*], sulfonamides [*sul1*, 3x, and *sul2*], macrolides [*ere(A)*], phenicols [*catA2*], tetracyclines [*tet(D)*], trimethoprim (*dfrA19*), and quaternary ammonium compounds (*qacΔE*; two copies). The *mcr-9.1* in p1-va18651 was flanked upstream by an IS903 and downstream by an IS26, identical to surroundings of previous studies (Li et al., 2020; Tyson et al., 2020; Macesic et al., 2021). An IncHI1-type replication protein, a transfer–conjugation system, and a toxin–antitoxin system were predicted on the plasmid backbone (**Figure 2A**). Using BLAST searching on GenBank, the p1-va18651 has significant homology to known plasmids of the *E. hormaechei* strain AR_0365 and *Salmonella enterica* strain CVM N23023 with identity >99.98% (**Table 3**). Remarkably, a homologous sequence (51% query and 99.99% identity) was also present in the chromosome of *Salmonella enterica* subsp. *enterica* serovar Heidelberg strain NY-N14748.

**Table 3:** Examples of sequences highly related to the plasmids of *Enterobacter xiangfangensis* va18651.

Plasmid	Query Length(bp)	Query Coverage (%)	Identity (%)	Homologies	Acc. length (bp)	Accession no.
p1-va18651 (mcr-9)	307,415	91 82 51	100 99.98 99.99	p-unnamed1 of strain AR_0365 pN53023 of strain CVM N23023 Chromosome of strain NY-N14748	328,871 339,705 4,984,436	CP027144 CP049311 CP048926
p2-va18651 (*bla*_KPC-2_)	79,326	100 100 100 100	100 100 100 100	pCP13069-KPC2 pCF08698-KPC2 pCF13141-KPC2 pKV30046-KPC2	78,021 78,021 78,021 78,023	VKMZ01000118.1 VKMD01000077.1 VKMY01000050.1 JAFHMT000000000
p3-va18651 (*bla*_OXA-48_)	63,589	100 100 100	100 100 100	pOXA-48_1639 pACV-OXA-48 p2247421	63,589 63,589 63,589, CP086451	LR025105, CP045727
p4-va18651	154,302	88	99.99	p-unnamed1 of *Enterobacter* strain EB_P6_L3_02.19	166,898	CP043854

The *bla*_KPC-2_-harboring plasmid p2-va18651 belonged to the plasmid incompatibility group IncN and was a member of the type pMLST15. It was 79,326 bp in size and had a GC content of 52.7% with 100 CDSs predicted. The p2-va18651 contained 14 different ARGs of diverse classes including β-lactams (*bla*_KPC-2_, *bla*_TEM-1B_, and *bla*_OXA-1_), aminoglycosides [*aac(II)-3d*, *strA*, and *strB*], fluoroquinolones [*aac(6´)-Ib-cr* and *qnrB2*], sulfonamides (*sul1*, two copies), macrolides [*mph(A)*], phenicols (*catB3*), rifampicin (*ARR-3*), trimethoprim (*dfrA19*), and quaternary ammonium compound resistances (*qacEΔ_1*). The p2-va18651 exhibited a unique genetic environment surrounding the *bla*_KPC-2_ gene, being identical to that of a persistent and promiscuous IncN[pMLST15] plasmid from different species in previous studies (Yao et al., 2014; Yao et al., 2021). Comparison analysis revealed that p2-va18651 was virtually identical to plasmids obtained from *Enterobacterales* species isolates from human, food, and environment that have been in circulation for long time in healthcare and environmental settings in Germany hospitals (see pCP13069-KPC2, pCF08698-KPC2, pCF13141-KPC2, and pKV30046-KPC2) as well as to plasmids from isolates obtained within the SurvCARE-Project (unpublished data). The plasmid p2-va18651 only differed from them with an IS*5702* insertion in its multidrug cargo region (**Table 3** and **Figure 2B**). The backbone of the p2-va18651 contained a conjugation system (**Figure 2B**).

The *bla*_OXA-48_-carrying plasmid p3-va18651 was an IncL-plasmid with 63,589 bp length and a GC content of 51.2% and exhibited 85 predicted CDSs (**Figure 2C**). The plasmid p3-va18651 carried the *bla*_OXA-48_ carbapenemase gene, which is embedded within a Tn*1999* flanked by an IS*1999* transposase at both ends. It contained a typical IncL backbone comprising features of replication (*repA* and *repC*), maintenance, and stabilization (plasmid partition *parA*, *parB*, toxin–antitoxin system *pemL*, *pemK*, *ssb*, anti-restriction *kicA*, *korC*, and *eexA*) as well as transfer–conjugation machinery (*trbABCN* and *traHIJKLMNOPQRUWXY*) and mobility cassette-encoding *mobA* and *mobB* (**Figure 2C**). There were more than 80 sequence entries of strains from the species *Escherichia coli*, *Klebsiella pneumoniae*, *Citrobacter freundii*, *Serratia marcescens*, and *Raoultella ornithinolytica* in the NCBI database that were 100% identical to p3-va1865, suggesting promiscuous transmissibility (**Table 3**).

The fourth plasmid, p4-va18651, was an IncFII-B(pECLA)-type plasmid of 154,302 bp in size and with a GC content of 51.5%, which encoded 174 predicted CDSs (**Figure 2D**), but no known antibiotic resistance genes. p4-va18651 harbored a transfer gene cluster, indicating that it was a conjugative plasmid. A similar plasmid with 88% coverage and an identity of 99.99% (166,898 bp) was found from an *E. hormaechei* strain EBp6-L3 (ST88) isolated in the UK 2019 (Accession no. NZ_CP043854.1).

### Virulence factors and heavy metal and metalloid resistome of va18651

The strain va18651 was classified as *Enterobacter* ST88 based on the *in silico* MLST typing scheme. Diverse virulence genes for the lipopolysaccharide and capsular polysaccharide, iron acquisition (*iroBCDEN*, *ent/fep/fes*, *sitABCD*, *iut*, and *fur*), and a Type VI secretion system, together with motility genes, Type I fimbria, and Type IV pili were encoded on the chromosome (**Figure 1**). The PhoPQ two-component regulator and its negative inhibitor *mgrB* that plays a global regulatory role in antibiotic susceptibility, physiology, stress adaptation, and virulence were intact. The presence of a number of previously determined virulence determinants indicated that va18651 carried a high pathogenic potential.

Notably, two of the four plasmid harbored heavy metal and metalloid resistance genes. These included resistance gene clusters for tellurium (*terA*, *terB*, *terC*, *terD*, *terE*, *terX*, *terY*, *terW*, and *terZ*), cadmium (*cadA* and *cadR*), mercury (*merA*, *merC*, *merD*, *ere*, *merP*, *merR*, and *merT*), copper (*pcoE* and *pcoS*) and arsenate (*arsB*, *arsC*, *arsH*, and *arsR*) in the p1-va18651, as well as the gene cassettes of silver (*silA*, *silB*, *silF*, *silC*, *silR*, *silS*, and *silP*), copper (*pcoA*, *pcoB*, *pcoC*, *pcoD*, *pcoE*, *pcoS*, and *pcoR*) and arsenate (*arsA*, *arsB*, *arsC*, *arsD*, and *arsR*) in p4-va18651 (**Figure 2**).

## Discussion

In a study between 2017 and 2019, we collected carbapenem-resistant *Enterobacterales* isolates (CRE) obtained from patients covering the entire state of Hessen in Germany. Among these, isolate va18651 was found to harbour two carbapenemase-encoding genes, bla_KPC-2_ and bla_OXA-48_, and the mobile colistin resistance *mcr-9* gene. This combination was unique and not found in the *Enterobacter* species collection. We also screened 3,246 genomes of *Enterobacter* strains available from the public database to examine if the combination of these genes had been previously reported. The analysis revealed only nine isolates that harbored the combination of two carbapenemases and *mcr-9*; eight of these isolates were classified as members of the species *E. xiangfangensis*. While the OXA-48 is frequently detected in other *Enterobacterales* species, such as *Klebsiella* and *E. coli*, this is the first time we observed the OXA-48 determinant in the genus *Enterobacter*.

The isolate va18651 was initially identified as a member of ECC by MALDI TOF. Due to taxonomic conflicts and the low discriminatory power of MALDI TOF, *Enterobacter* species may not be correctly identified to the species level (Pavlovic et al., 2012; Godmer et al., 2021). Using recommended genome sequence-based approaches (Chun et al., 2018), the isolate va18651 was identified as *E. xiangfangensis* (also listed as *E. hormaechei* subspecies *xiangfangensis*) (Sutton et al., 2018; Wu et al., 2020). *Enterobacter* isolates have been reported to be present in the cervix of healthy individuals (Larsen and Monif, 2001; Amabebe and Anumba, 2018) and constitute a risk factor for the urinary tract infection and premature births.

The pathogenicity and virulence gene repertoire of *Enterobacter* are not well understood (Sanders and Sanders, 1997). Very few studies that examined the virulence capabilities of *Enterobacter* species in a mouse model of infection are available (Paauw et al., 2009; Krzymińska et al., 2010; Pati et al., 2018). *Enterobacter* species belonging to MLST sequence type 88 (ST88) have been reported from sporadic human and animal clinical cases as well as from outbreaks (Jia et al., 2018; Börjesson et al., 2019; Tian et al., 2020). To compare va18651 to other ST88 isolates distributed worldwide, we retrieved genomes of 21 ST88 isolates. Interestingly, 8 of 21 (38%) ST88 isolates carried *mcr-9* genes on an identical IncHI2 plasmid (**Figure 1A**). A total of 10 (47.7%) isolates carried at least one carbapenemase (*bla*_KPC-2_ and *bla*_VIM-1_, *bla*_KPC-18_ and *bla*_NDM-1_). These data indicated that carbapenemases and *mcr-9* are relatively common to ST88. These data urge further systematic studies with the clonal type ST88 in order to validate the common association of carbapenemases and *mcr-9.* The presence of an ST type present in a wide range of hosts and in different environmental settings as reflected by its carriage of carbapenemase-encoding genes of different types such as *bla*_KPC-2_ and *bla*_OXA-48_ suggest the emergence of a successful clone transcending species and environmental barriers.

While it is common to observe plasmids of between 10 kb and 500 kb in individual isolates of *Enterobacterales*, the plasmidome of va18651 was, at 604,632 bp, unusually large (Stephens et al., 2020; Darphorn et al., 2021). Highly identical *bla*_KPC-2_-harboring IncN plasmids were reported earlier in Germany (Yao et al., 2014; Becker et al., 2018; Yao et al., 2021). The *bla*_OXA-48_ plasmid observed was identical to the OXA-48-encoding IncL plasmid, which is reported globally. IncL/M plasmids are an emerging threat as they represent a current source of class D carbapenemases and are responsible for the worldwide distribution of *bla*_CTX-M_. This *bla*_OXA-48_ IncL plasmid was reported from diverse *Enterobacterales*, suggesting that the p2-va18651 and p3-va18651 and the respective encoded antimicrobial resistances were acquired by horizontal transmission. The environment surrounding *mcr-9.1* associated with the IS*903B* of p1-va18651 was identical with those identified in both chromosomes and IncHI1 plasmids of previous studies (Tyson et al., 2020), indicating an *mcr-9* acquisition mediated by mobile genetic elements. In total, the isolate va18651 carried 26 different antibiotic resistance genes, each present in one to four copies, and was distributed among 10 classes including β-lactams, aminoglycosides, fluoroquinolone, macrolides, phenicol, rifampicin, tetracycline, trimethoprim, and quaternary ammonium compound resistance gene cassettes (*qacEΔ_1*). These genes mediate resistance to a broad range of antibiotics that correspond to the drug and detergent resistance phenotypes.

There are few reports of co-carriage of two carbapenemase classes such as KPC-2 and OXA-48 in *K. pneumonia*e ST11 in Taiwan and Egypt (Wang et al., 2019; Yang et al., 2022) and KPC-2 and NDM-1 in the *Enterobacter* ST88 strain from Colombia (Accession No. SRR3110109) as well as VIM with OXA-48 in *E. xiangfangensis* isolates (Tyson et al., 2020), but there are currently no reports on the co-existence of KPC-2 and OXA-48 carbapenemases together with a colistin resistance-encoding gene *mcr-9.1*.

*Enterobacter* species carry an *arnBCADTEF* gene cassette, which has shown to be responsible for the colistin heteroresistance (Kang et al., 2019). The presence of this gene cassette on the chromosome of va18651 encouraged us to determine the colistin heteroresistance capability. As heteroresistance is an incidental phenomenon, the colistin resistance may or may not be detected in routine laboratories. Nevertheless, such heteroresistance capability is important from a clinical perspective as it results in the failure of the antibiotic therapy (Band and Weiss, 2019; Band et al., 2021). Previous studies indicate that the *mcr-9* gene is not induced naturally, and expressed only from artificial promoters or in presence of colistin (Carroll et al., 2019; Kieffer et al., 2019). Thus, while the *mcr-9* gene is functional, the conditions that induce its activity are not known.

While va18651 carried a high number of different ARGs on plasmids (p1-va18651, p2-va18651, and p3-va18651), the co-occurrence of a higher number of heavy metal resistance genes (HGR) on the plasmids p1-va18651 and p4va18651 was remarkable. Such co-occurrence of ARG and HRG has been recognized for a long time (Foster, 1983), but this coexistence is poorly understood (Li et al., 2017). Nevertheless, it is likely to provide it with additional survival and adaptive properties in ecological niches within hospital settings.

The *E. xiangfangensis* isolate va18651 carries a unique combination of risk factors, i.e., virulence genes on the chromosome together with a large number of antibiotic and heavy metal/metalloid resistance genes on plasmids. The presence of multiple plasmids harboring highly mobilizable genetic platforms also provide a “sink” and reservoir for fueling the accelerated dispersion of multiple ARGs, thereby paving the way in creating antimicrobial resistant (AMR)-hypervirulent vectors that could spread easily, expanding the incidence of hard-to-treat infections with fatal outcomes.

## Data availability statement

The data presented in this study are available through the Bioproject PRJNA837392 in the NCBI databases.

## Author contributions

YY and TC conceptualized the manuscript. YY and SD performed the bioinformatic analysis and wrote the draft of the manuscript. JF, JS, SD and CI performed the phenotyping and WGS genome sequencing. TC, SD and YY prepared the final manuscript with inputs from all Co-authors. All authors contributed to the article and approved the submitted version.

## Funding

This work was supported by the German Federal Ministry of Education and Research (BMBF) within the German Center for Infection Research (DZIF)/grant numbers, 8032808811, 8032808818, and 8032808820 to TC/CI, and 031L0209B, Deep-iAMR to TC.

## Conflict of interest

The authors declare that the research was conducted in the absence of any commercial or financial relationships that could be construed as a potential conflict of interest.

## Publisher’s note

All claims expressed in this article are solely those of the authors and do not necessarily represent those of their affiliated organizations, or those of the publisher, the editors and the reviewers. Any product that may be evaluated in this article, or claim that may be made by its manufacturer, is not guaranteed or endorsed by the publisher.
